# Robotic-assisted thoracic surgery training in France: a nation-wide survey from young surgeons

**DOI:** 10.1093/icvts/ivae115

**Published:** 2024-06-18

**Authors:** Hugo Clermidy, Guillaume Fadel, Benjamin Bottet, Yaniss Belaroussi, Maroua Eid, Elsa Armand, Jean-Marc Baste, Pierre-Benoit Pages, Alex Fourdrain, Charles Al Zreibi, Leslie Madelaine, Gabriel Saiydoun, AJCTCV, Chloé Bernard, Marie Jungling, Hayat Aiouaz, Solenne Vasse, Antoine Buschiazzo, Paul Borchiellini, Johann Cattan, Saadé Saade

**Affiliations:** Department of Thoracic Surgery, Lung and Heart-Lung Transplantation, Louis Pradel Hospital, Hospices Civils de Lyon, Lyon, France; Association of Thoracic and Cardiovascular Surgeon in Training, Association des Jeunes Chirurgiens Thoracique et Cardio-Vasculaire, Paris, France; Association of Thoracic and Cardiovascular Surgeon in Training, Association des Jeunes Chirurgiens Thoracique et Cardio-Vasculaire, Paris, France; Department of Thoracic Surgery and Heart-Lung Transplantation, Université Paris-Saclay, Marie Lannelongue Hospital, Le Plessis Robinson, France; Association of Thoracic and Cardiovascular Surgeon in Training, Association des Jeunes Chirurgiens Thoracique et Cardio-Vasculaire, Paris, France; Department of General and Thoracic Surgery, CHU Rouen, Rouen, France; Association of Thoracic and Cardiovascular Surgeon in Training, Association des Jeunes Chirurgiens Thoracique et Cardio-Vasculaire, Paris, France; Department of Thoracic Surgery, Haut-Leveque Hospital, University of Bordeaux, Bordeaux, France; Association of Thoracic and Cardiovascular Surgeon in Training, Association des Jeunes Chirurgiens Thoracique et Cardio-Vasculaire, Paris, France; Department of Cardiac Surgery, University of Angers, Angers, France; Association of Thoracic and Cardiovascular Surgeon in Training, Association des Jeunes Chirurgiens Thoracique et Cardio-Vasculaire, Paris, France; Department of Thoracic Surgery, Lung Transplantation and Esophageal Diseases, North Hospital, Marseille, France; Department of General and Thoracic Surgery, CHU Rouen, Rouen, France; Cardiovascular and Thoracic Surgery Department, Dijon University Hospital, Dijon, France; Department of Thoracic Surgery, Lung Transplantation and Esophageal Diseases, North Hospital, Marseille, France; Association of Thoracic and Cardiovascular Surgeon in Training, Association des Jeunes Chirurgiens Thoracique et Cardio-Vasculaire, Paris, France; Department of Thoracic Surgery, Georges Pompidou European Hospital APHP, Paris, France; Association of Thoracic and Cardiovascular Surgeon in Training, Association des Jeunes Chirurgiens Thoracique et Cardio-Vasculaire, Paris, France; Cardiovascular and Thoracic Surgery Department, Dijon University Hospital, Dijon, France; Association of Thoracic and Cardiovascular Surgeon in Training, Association des Jeunes Chirurgiens Thoracique et Cardio-Vasculaire, Paris, France; Department of Cardiac Surgery, Pitié Salpétrière University Hospital, Sorbonne University, APHP, Paris, France; Department of Cardiac Surgery, Henri Mondor University Hospital, APHP, Créteil, France

**Keywords:** Simulation, Robotic-assisted thoracic surgery, RATS, Resident training

## Abstract

**OBJECTIVES:**

Evaluate theoretical and practical training of thoracic surgeons-in-training in robotic-assisted thoracic surgery (RATS) in France.

**METHODS:**

A survey was distributed to thoracic surgeons-in-training in France from November 2022 to February 2023.

**RESULTS:**

We recruited 101 thoracic surgeons-in-training (77% response rate). Over half had access to a surgical robotics system at their current institution. Most (74%) considered robotic surgery training essential, 90% had attended a robotic procedure. Only 18% had performed a complete thoracic robotic procedure as the main operator. A complete RATS procedure was performed by 42% of fellows and 6% of residents. Of the remaining surgeons, 23% had performed part of a robotic procedure. Theoretical courses and simulation are well developed; 72% of residents and 91% of fellows had undergone simulation training in the operating room, at training facilities, or during congress amounting to <10 h (for 73% of the fellows and residents), 10–20 h (17%), 20–30 h (8%) or >30 h (3%). Access to RATS was ≥1 day/week in 71% of thoracic departments with robotic access. Fellows spent a median of 2 (IQR 1–3) semesters in departments performing robotic surgery. Compared with low-volume centres, trainees at high-volume centres performed significantly more complete robotic procedures (47% vs 13%; *P* = 0.001), as did fellows compared with residents.

**CONCLUSIONS:**

Few young surgeons perform complete thoracic robotic procedures during practical training, and access remains centre dependent. Opportunities increase with seniority and exposure; however, increasing availability of robotic devices, theoretical formation, and simulation courses will increase opportunities.

## Graphical abstract

**Figure:**
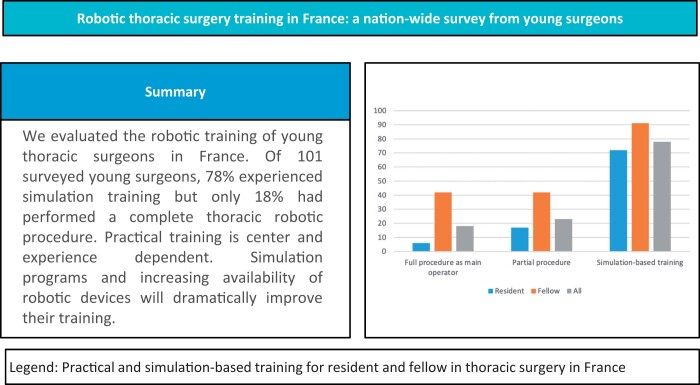

## INTRODUCTION

The 1st documented thoracic robotic programme in Europe was launched in Italy by Professor Franca Melfi in 2000 [1]. In France, robotic-assisted thoracic surgery (RATS) was introduced more recently. Robotic surgery is expanding rapidly in all specialties. Intraoperative complications during robotic thoracic surgery are rare but potentially life-threatening [2]; thus, acquiring proficiency is crucial. Recently, a working panel comprising members of the European Society of Thoracic Surgeons and European Association for Cardio-Thoracic Surgery suggested a standardized training curriculum for robotic thoracic surgery [3], with a baseline evaluation, e-learning modules, simulation-based training (including virtual-reality simulation, dry lab and wet lab), and robotic bedside observation. Advanced robotic training should include e-learning on index procedures with access to a video library of robotic procedures, modular console training to index procedure, transition to full-procedure training with a proctor, and final evaluation of the submitted video. Key opinion leaders conducted a survey on simulation-based training to integrate findings into the thoracic surgical curriculum, identifying only port placement, docking and undocking as technical procedures for RATS [4].

Access to robotic surgery differs significantly between and within countries [5–7]. Gandhi *et al.* [5] reported that most respondents had limited (31%) or no (22%) access to RATS, and 52% did not consider RATS for their future curriculum, highlighting the lack of training opportunities. Despite the clear necessity for a standardized robotic surgery curriculum, few programmes exist [3, 5].

In France, thoracic surgery training follows a structured timeline and framework. Medical students choose a specialty and location based on National Medical Examination results, completed at the end of the 6th year of studies [5]. The quality and experience of residents’ training in robotic surgery is dependent on the equipment and expertise of the chosen institution. The residency programme spans 6 years: a foundation year, 3 years of progressively detailed and in-depth training, then 2 years honing accountable autonomy and skill consolidation as ‘junior doctors’. Following residency, fellowships (2–4 years) enable skill refinement, ultimately leading to full autonomy in thoracic surgery.

Access and availability of robotic devices and RATS varies between institutes, with some sharing the robotic device between surgical specialties. Furthermore, senior thoracic surgeons who completed residency training before RATS was developed have to learn new surgical systems before training residents, which may impact young surgeons’ training.

To enhance residents’ training, the French Society of Thoracic and Cardio-Vascular Surgery (SFCTCV) has implemented educational strategies including virtual-reality simulation, wet-lab simulation and immersion courses at expert robotic centres to optimize resident education in preparation for autonomous practice in thoracic surgery. Despite these initiatives, some residents and fellows might be not proficient in RATS. Thus, the present study was conducted to evaluate practical and theoretical robotic surgery training for thoracic surgeons in France, and to assess their perceptions and expectations of RATS training, using a national web-based survey.

## MATERIALS AND METHODS

### Ethical statement

This study was approved by the institutional review board of the SFCTCV (IRB 00012919). The results are reported according to Strengthening the Reporting of Observational Studies in Epidemiology (STROBE) guidelines [8].

### Survey

Between November 2022 and February 2023, the *Association des Jeunes Chirurgiens Thoracique et Cardio-Vasculaire* sent a descriptive survey to thoracic surgeons-in-training. The survey was approved by the SFCTCV. Surveys were anonymous, voluntary and web-based, and were sent to residents in the 2nd–6th year, specializing in only thoracic surgery (*n* = 40) or in undifferentiated thoracic or cardiac surgery (*n* = 53), as well as thoracic fellows up to their 4th year of fellowship (*n* = 39). First-year residents were excluded due to insufficient surgical experience. Residents specializing solely in cardiac surgery were excluded. Incomplete forms (<75% of the total items) were excluded. Data were collected via online questionnaire (Survey Monkey, San Mateo, CA, USA) sent via email, using our national demographic database. Two reminder emails were sent during the study period with the support of the SFCTCV to improve response rates. Multiple reminders were sent to residents during the biannual seminar and annual congress.

The questionnaire included 28 items on demographics, exposure to RATS, experience in bedside assist, and performance of partial/complete robotic procedures as well as respondents’ perceptions and expectations regarding robotic training and their perceived ability to perform robotic surgery independently. The questionnaire is included in the Supplementary material.

### Training programmes

The SFCTCV leads several simulation and training programmes. French Bootcamp—held at the Rouen Medical Training Centre—comprises 2 days of immersion in a surgical-simulation bootcamp. Third year residents practice basic skills, thoracoscopic and robotic skills, and communication skills. The SFCTCV also offers 2 wet-lab experiences (involving Pulse for Practice technology by Simedys to re-create perfused cadavers) during early and late residency, to develop open skills and thoracoscopic/robotic skills, respectively. Recently, the SFCTCV organized a RATS tour for junior doctors and fellows based on a ‘*Tour de France*’ of expert centres, with 2 days immersion at each centre.

### Definition of surgical procedures and volume centres

For this study, procedures were considered complete when the resident or fellow performed over 75% of the surgery as the main operator. A senior surgeon could remain at the other robotic console for guidance. An intervention was considered partial when the resident or fellow took the lead at the robot console to perform any part of the procedure (e.g. vessel dissection and stapling, lymph-node dissection, dissecting the triangular ligament). High- and low-volume centres were defined as centres where RATS was performed ≥2 or ≤1 day/week, respectively. Median numbers of semesters in departments with RATS access were calculated for fellows only because residents had not finished their whole training.

### Statistical analyses

Statistical analyses were performed using Prism 10.0.3 (GraphPad Software, San Diego, CA). Categorical variables were analyzed using Chi^2^ test or Fisher’s exact test for small samples. Continuous data were compared using the Student’s *t*-test (parametric test) or Mann–Whitney *U*-test (non-parametric test). Logistic regression was used to correlate exposure to RATS and complete procedure performed. Statistical significance was accepted as *P* < 0.05.

## RESULTS

### Demographics and robotic exposure

**Figure 1:**
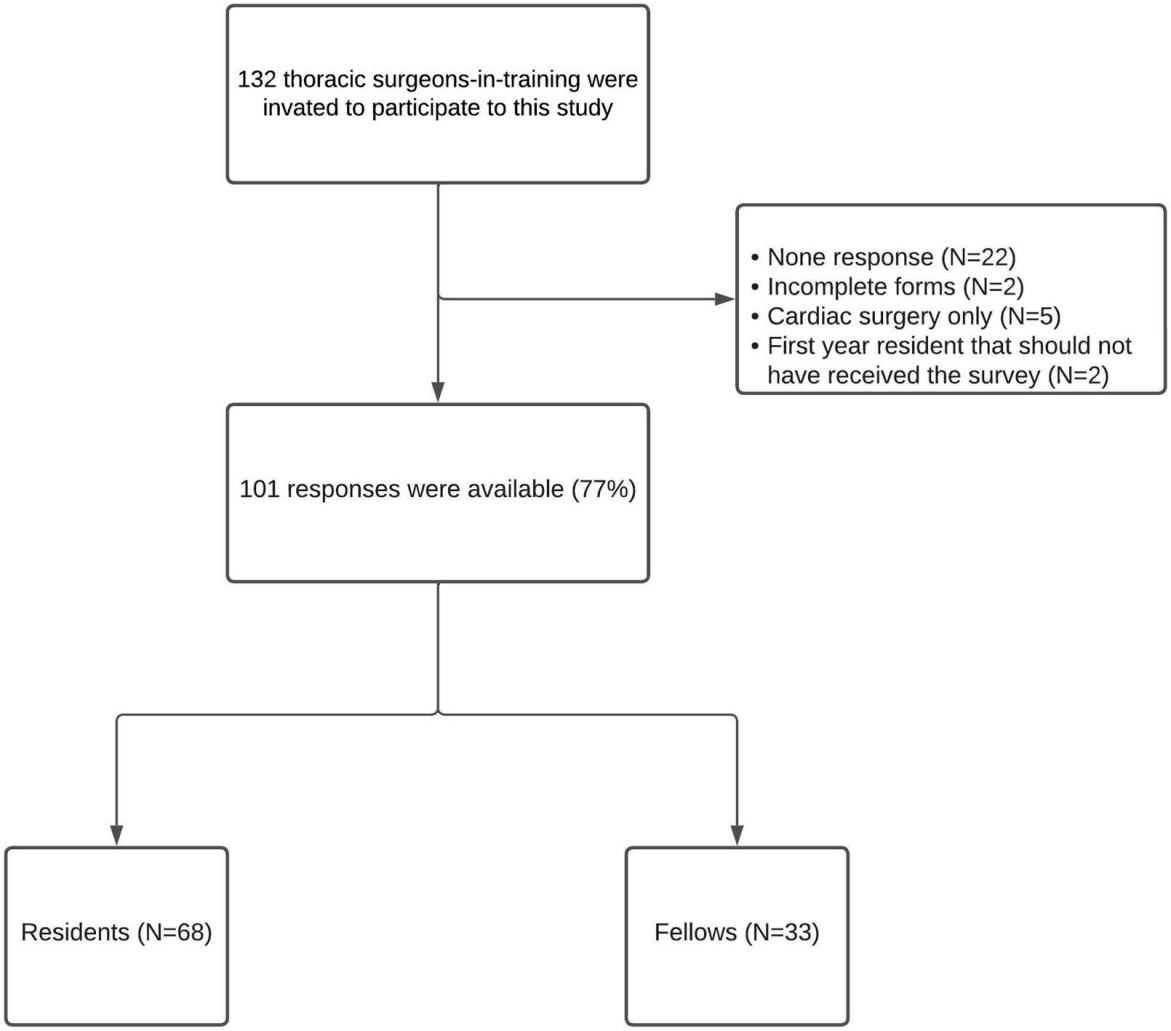
‘Flow chart’ of the survey.

We recruited 132 thoracic surgeons-in-training and received 110 responses. Two were excluded due to incomplete responses and 5 were excluded due to interest in cardiac surgery only despite initially declaring undifferentiated cardio-thoracic training. Of the 101 who were finally enrolled, 39% (39/101) were women. We invited 93 residents to participate; 73% (68/93) responded (11, 20, 16, 14 and 7 residents in the 2nd, 3rd, 4th, 5th and 6th year of residency, respectively). Of 39 fellows, 85% (33/39) responded (5, 16 and 20 in the 1st, 2nd and third-or-higher year of fellowship, respectively) (Fig. 1).

**Figure 2:**
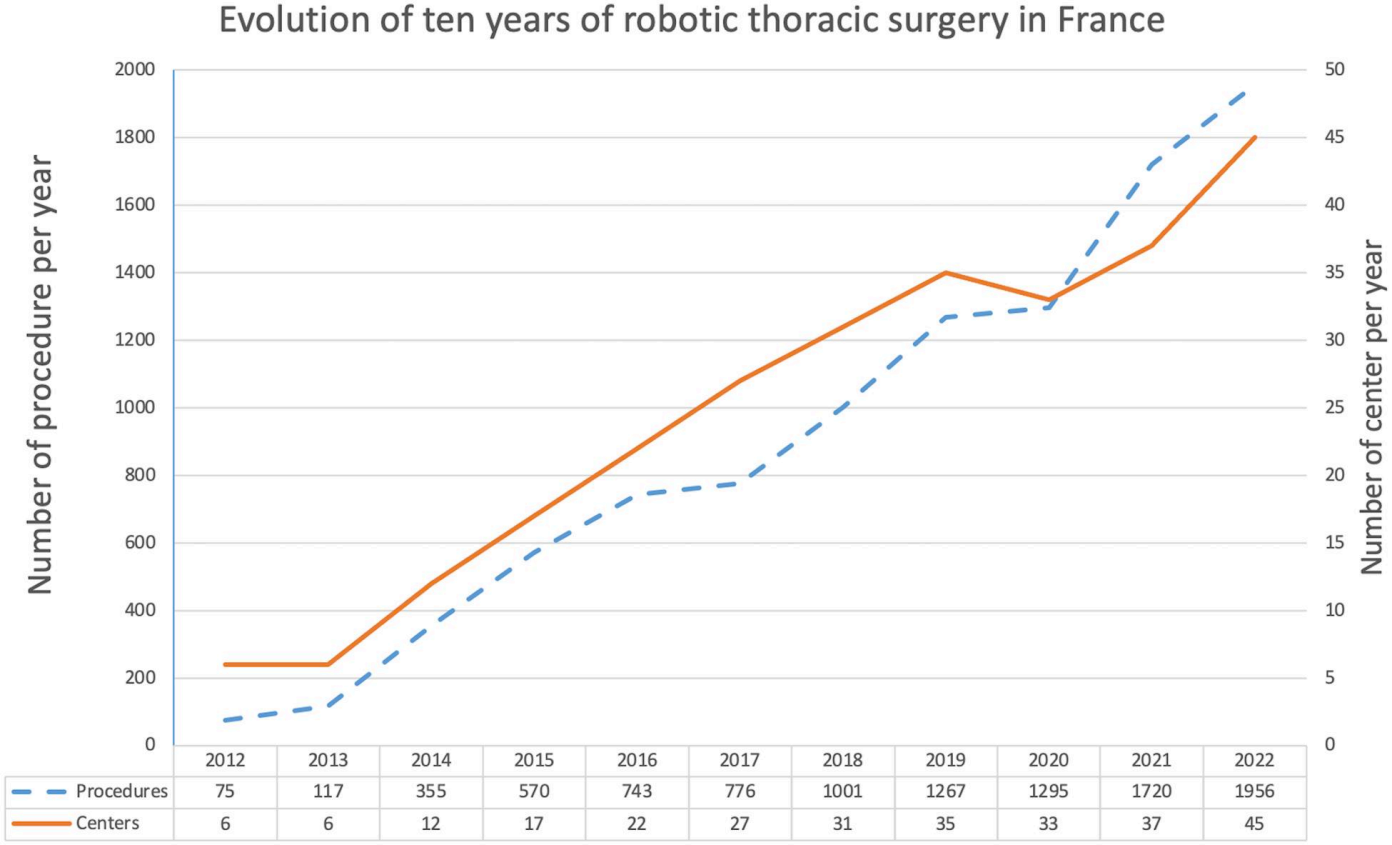
Evolution of robotic surgery (number of procedure, number of centres) during the last 10 years.

Over half of the thoracic surgeons-in-training had access to surgical robotics systems at their training institutions (Table 1). Eleven institutions did not perform RATS routinely. The most common frequency for performing RATS was 1 day/week (70%, 41/58). Only 2 departments from teaching hospital (Dijon and Rouen) reported robotic access 3 days/week. Fellows spent a median of 2 [interquartile range 1–3] semesters in a department with access to robotic surgery during their residencies. Of thoracic surgeons-in-training, 79% (80/101) spent at least 1 semester in a department using robotic surgery. At the time of the survey, 45 centres (teaching hospitals and private clinics) in France had robotic access, with increasing numbers of robotic procedures (Fig. 2).

**Table 1:** Summary of practical and theoretical training according to the resident or fellow status

	Resident (*n* = 68)			Fellow (*n* = 33)			All (*n* = 101)			*P*-value
Practical training										
Institutional RATS access, yes, *n* (%)		38	(56)		20	(60)		58	(57)	0.65
Complete procedure, yes, *n* (%)		4	(6)		14	(42)		18	(18)	<0.0001
Partial procedure, yes, *n* (%)	n/64	11	(17)	n/19	8	(42)	n/83	19	(23)	0.03
Bedside assistant active role, yes, *n* (%)	n/57	52	(91)	n/32	32	(100)	n/89	84	(94)	0.15
Theoretical training										
Simulation training, yes, *n* (%)		49	(72)		30	(91)		79	(78)	0.04
Simulation > 10 hours, yes, *n* (%)	n/49	9	(18)	n/30	12	(40)	n/79	21	(21)	0.03
Sufficient simulator access perception, yes, *n* (%)	n/49	10	(20)	n/30	13	(43)	n/79	23	(29)	0.04
Wet-lab training, yes, *n* (%)		7	(10)		11	(33)		18	(18)	0.01

### RATS practical training

The vast majority (90%, 91/101) of surgeons-in-training had already attended a robotic procedure and 18% had performed a complete thoracic robotic procedure as the main operator (Table 1). Few residents (1 in their 4th, 2 in their 5th and 1 in their 6th year) had performed a complete procedure; the procedures performed were lobectomy (*n* = 1), mediastinal mass excision (*n* = 1) and wedge resection (*n* = 2). Fourteen fellows had performed a complete robotic procedure, most were in their 3rd or 4th year of residency (*n* = 12). All except 1 fellow (who was in their 1st year) had performed a major pulmonary or mediastinal resection. Half (7/14), 28.6% (4/14) and 21.4% (3/14) had performed ≤5, 6–20 and ≥20 major robotic procedures, respectively. The rate of complete RATS procedure increased significantly with seniority (fellow vs resident) (odds ratio [OR] 11.8, 95% confidence interval [CI] 3.7–35, *P* < 0.0001). Trainees at high-volume centres performed significantly more complete robotic procedures compared with low-volume centres (47% vs 13%) (OR 6, 95% CI 1.8–18.7, *P* = 0.001). The number of semesters fellows spent during residency in a department performing RATS was significantly associated with complete RATS procedures (OR 1.6, 95% CI 1.12–2.61, *P* = 0.02).

Almost a quarter (23%, 19/83) of surgeons-in-training who had not performed a complete procedure had performed part of a robotic procedure (Table 1). Thus, 63% (64/101) never performed any part of a surgical procedure at the master console. However, the majority of residents who attended RATS were active assistants.

### RATS simulation and theoretical courses

Most respondents (97%) were aware of virtual-reality robotic simulators; the majority had experienced it (Table 1). Most residents and fellows had experienced the simulator, primarily in the operating room (55%, 46/83), training facilities (23%, 19/83), during congress (12%, 15/83) or in industrial facilities (4%, 3/83). Simulation training time was <10, 10–20, 20–30 and >30 h in 73% (58/79), 17% (13/79), 8% (6/79) and 3% (2/79) of cases, respectively.

Access to RATS wet-lab training (perfused cadavers or animal models) was limited, but access increased with seniority (Table 1).

### Perceptions and expectations of RATS training

The majority (74%) of surgeons-in-training considered robotic surgery training fundamental for their future, 25% considered it useful, 1% considered it useless. Over half (59%) of residents and 49% of fellows did not consider themselves capable of performing a full RATS procedure independently at the end of training operator at the end of residency; 15% and 33%, respectively, considered themselves competent, while 25% of residents and 18% of fellows did not specify.

The median satisfaction score of RATS training was 2/5. Access to simulation training was considered insufficient by 71% of trainees. However, 67% of surgeons-in-training considered the ‘RATS tour’ useful, 10% were not interested, 16% had no opinion and 7% had already participated.

## DISCUSSION

Robotic-assisted surgery has become widespread in common general surgical procedures. Gaining expertise in robotic-assisted surgery is therefore becoming an essential part of surgical training. Our study highlights the heterogeneity of practical training among centres and age groups. While the high rate of RATS experience reported here reflects the recent increase in RATS-experienced surgeons, few had performed a complete RATS procedure as the main operator. While fellows were more experienced in complete RATS procedures, only around a third considered themselves capable of performing RATS independently. Thus, few surgeons-in-training in France are autonomous in RATS. However, the development and availability of simulation training for all young surgeons may address this.

Robotic surgery has been applied to thoracic surgery more recently than to other specialties. RATS is performed by 44% of thoracic surgery departments, and 12 new centres opened in the past 2 years, meaning that the paucity in training opportunities is decreasing; indeed, we found that most trainees had exposure to RATS during their residency. The earlier implementation of number of available surgical robotic systems in the USA may explain why 96% of residents have access to surgical robotics system at their training institution [9], which is almost double the rate of the present study. Overall, 63% of residents in the USA stated that they had participated in robotic surgical cases [10], with only 12% of residents in general surgery reporting no exposure to robotic surgery in 2022 [11].

A survey from the European Society of Thoracic Surgeons (ESTS) Robotic Working Group [5] highlighted the increasing adoption of RATS in Europe, but also emphasized significant gaps in training and curriculum integration across the continent. The importance of a structured, standardized training programme for thoracic robotic surgery in Europe has been acknowledged [12], and authors from Europe [3, 12], the USA [13, 14] and Australia/New Zealand [15] have expressed concerns about the lack of such programmes for various specialties [16]. In the USA, neither the American Association of Thoracic Surgery nor the Society of Thoracic Surgeons sponsors a widely accepted curriculum for residents, nor is robotic experience required to sit for American Board of Thoracic Surgery certification [17]. The European Society of Thoracic Surgeons offers robotic courses primarily dedicated to fellows. In France, our bootcamp is the only mandatory training programme for 3rd-year cardio-thoracic surgery residents, although it does not specifically focus on robotic surgery.

The low rate of RATS experience in our study somewhat conflicts with Gergen *et al.* [18], who reported that 66% of residents performed >50% of operations independently at the surgical console, and 33% performed >75% of the operation. However, Farivar *et al.* [10] reported that 63% of surgeons-in-training with a general surgery curriculum had participated in a surgical procedure, but fewer than 20% reported operating from the robotic console, in line with our findings that a minority of residents had performed a complete procedure of any kind.

Limitations to RATS training in France are multifactorial; 1st, in the early phases of residency, the curriculum requires that residents assist in open and video-assisted thoracoscopic surgery (VATS). Only then can they learn the basics of RATS (port placement, robot docking, changing instruments and removing lymph nodes or suture gauze roll). We found that the majority of residents are active bedside assistants, which is the 1st step of RATS training. Second, the choice of training centres during residency and fellowship is crucial. Performance of a complete RATS procedure was typically achieved by trainees at high-volume centres who had early exposure to RATS devices, demonstrating the centre-dependence of RATS training. Moreover, for many currently practicing thoracic surgeons, RATS developed many years after their own surgical residency training. With the increasing purchase of robotic device, the learning curve introduced due to ‘senior’ thoracic surgeons acquiring RATS skills later in their careers further inhibits the implementation of RATS training for younger surgeons. Young surgeons may find the lack of haptic feedback compared with VATS difficult to overcome, which could limit senior surgeons from more easily delegating some parts of the surgery. Together, these limitations could explain why we found the perception of RATS training to be low in France.

Virtual-reality robotic simulation is a cost-effective and accessible training method [19]. The present study supports the benefits of the free bootcamps and wet labs offered by the SFCTCV, which enable students to attend simulation workshops adapted to training requirements for thoracic and cardio-vascular surgery, and learn crisis-management and communication skills. It also offers virtual-reality robotic simulation, and junior doctors who attended the ‘RATS Tour’ are evaluated when performing RATS lobectomy on porcine models.

Wet-lab simulations are also undertaken in each phase of residency using the Pulse for Practice technology, during which younger resident are teamed with ‘confirmed’ residents or junior doctors. Experienced surgeons can also perform VATS or RATS lobectomy, then assist earlier residents with open lobectomies. Although only 10% and 33% of residents and fellows, respectively, performed RATS wet-lab lobectomies, this will increase with the integration of Pulse for Practice workshops into residencies and the growing interest in RATS over VATS. Worldwide, there are many training programmes available to surgeons-in-training, such as those developed by the American Association of Thoracic Surgery (AATS), which incorporate porcine models [20]. However, perfused cadavers are not common, thus representing a unique opportunity for young surgeons training in France. Similar to the ‘RATS Tour’, the ESTS offers the chance for fellows to visit multiple European expert centres in RATS through the ESTS robotic school [21].

Our study reveals that access to simulators did not result in significant time spent using the equipment, with many trainees considering access insufficient. Similarly, Shaw *et al.* [22] reported that, despite high availability of simulators, only 48% of residents used it for the required learning time, and 38% had used the simulator once a month at most. This may be due to the robotic console being in another building or hospital in some cases, as well as a lack of dedicated time for simulation training for young surgeons. Thus, early residents’ work routines need to be changed to accommodate regular simulation [23]. The Association des Jeunes Chirurgiens Thoracique et Cardio-Vasculaire (Association of thoracic and cardiovascular surgeons-in-training, AJCTCV) suggest the integration of a simulation session in the operative list (as a regular operation after finishing the operative list, for example) to ensure periodical theoretical training, with identified operators. Simulation-based workshops during conferences and annual meetings are also highly recommended [24].

Despite a lack of exposure in certain thoracic departments in France, we found that most thoracic surgeons-in-training felt training in RATS surgery is a fundamental part of training. In the USA, the Society of Thoracic Surgeons workforce on Thoracic Surgery Resident Issues Transition to Practice Task Force conducted a voluntary survey of thoracic surgeons-in-training, reporting that 56% of respondents lacked confidence in robotic surgery. However, learning robotic technology has been shown to be safer and more efficient than VATS, considering the functionalities of the robot [25]. The introduction of the Da Vinci (Intuitive Surgical, Sunnyvale, California) dual-console robotic surgical system offers the advantage of a master console for the proctoring surgeon and a secondary console for the proctored surgeon, allowing teacher and trainee to swap control of the robotic instruments via their respective consoles while sharing the same intracorporeal view and communicating via a microphone system. Teaching robotic surgical techniques should, therefore, not be feared by either the teacher or student. Indeed, Cerfolio *et al.* [26] demonstrated that robotic lobectomy can be taught safely by allowing surgeons-in-training to perform various surgical manoeuvers, without compromising outcomes for the patient. In some cases, implementing standardized protocols and procedures can reduce morbidity and mortality and simulation-based trials have demonstrated improvements in managing major crises within the operating room [27, 28]. A new approach in RATS, known as ‘French Lobectomy’, is currently being developed (article in press), which divides the pulmonary hilum into 5 distinct zones to provide a simplified and standardized framework for describing the 5 lobectomies. Implementing this standardized approach will greatly enhance communication between trainer and trainee, ensuring clear and effective knowledge transfer in robotic surgical training.

Expansion of RATS could lead to residents losing their open surgical skills, which will remain fundamental in the future when open surgery may be restricted to advanced (and difficult) cases, bleeding conversion and post chemo-immunotherapy surgery. Therefore, RATS training should not inhibit learning of basic open skills which, along with VATS skills, should be mastered before robotics skills. Ultimately, it is the responsibility of senior surgeons to transmit their open knowledge.

Robotic access is fundamental to young residents’ training, and RATS training in the future will likely be as common as VATS or open-surgery training. Simulation training should be mandatory before practical training, with bedside assistance as the 1st step. Practical training could increase gradually during residency, with the dual console enabling fully trained senior surgeons to gradually increase delegation to the resident. According to experience, the following steps could be delegated: Non-traumatic manipulation of the lung, coagulation instruments through simpler procedures such as triangular-ligament section and lymph-node dissection, robotic stapling during management of pneumothorax and wedge resections, dissection of the pulmonary hilum and vessels up to full lobectomy. Gradual increase in the complexity of procedures could allow trainees to achieve segmentectomies, under the supervision of a senior surgeon at the dual console. Two recent multicentre randomized trials in 2023 [29, 30] will probably encourage a growth in RATS due to the increased surgical precision compared with VATS, use of articulated instruments, and 3D vision. These advantages should reduce the learning curve of sublobar resection for young surgeons. However, regular trainer/trainee exchange via a validated technical skill assessment will remain essential for successful training, along with reviews of intraoperative recordings of RATS procedures.

## CONCLUSION

Most surgeons-in-training consider training in robotic surgery to be essential, and desire greater exposure to robotic surgery and virtual simulators. However, the heterogeneity and centre dependency of practical training presents significant challenges to thoracic surgeons-in-training in France. Therefore, the quality of training varies and not all surgeons can act as independent operators in robotic surgery. Implementing dedicated programmes, such as the Bootcamp and wet-lab offered by our society, will increase accessibility of virtual training and wet-lab simulation. The development of standardized protocols and procedures for robotic surgery, accompanied with theoretical training, will ensure clear and effective knowledge transfer between trainer and trainee.

## Supplementary Material

ivae115_Supplementary_Data

## Data Availability

The data underlying this article will be shared on reasonable request to the corresponding author.

## Notes

Presented at the annual meeting of the French Society of Thoracic and Cardio-Vascular Surgery, 1 June 2023, Nantes, France.

## ABBREVIATIONS

AJCTCV: Association des Jeunes Chirurgiens Thoracique et Cardio-Vasculaire (Association of thoracic and cardiovascular surgeons-in-training)
RATS: Robotic-assisted thoracic surgery
SFCTCV: Société Française de Chirurgie Thoracique et Cardio-Vasculaire (French Society of Thoracic and Cardio-Vascular Surgery)
VATS: Video-assisted thoracoscopic surgery
