# Supplementary material for: Robotic-assisted thoracic surgery training in France: a nation-wide survey from young surgeons
Source: Interdiscip Cardiovasc Thorac Surg. 2024 Jun 18;39(1):ivae115. doi: 10.1093/icvts/ivae115 (PMC11219244; doi:10.1093/icvts/ivae115)
Supplement: ivae115_Supplementary_Data [file ivae115_supplementary_data.docx]

**Annex 1 - Questionnaire:**

| 1.Who are you? | Female  Male |
| --- | --- |
| 2.If you are a resident, in which year of residency do you are?  If you are a fellow, in which year of fellowship do you are ? | (Free answer) |
| 3.What is your residency institution (chose at the end of the national medical exam)? | (Free answer) |
| 4.If you are a Junior Doctor or a fellow, what is your current institution | (Free answer) |
| 5.Which type of practice do you plan in the future ? | Public hospital  Private clinic  No opinion |
| 6.Have you ever attended RATS? | Yes  No |
| 7.Does your institution have a robotic device ? | Yes  No |
| 8.Is surgical robot available for thoracic surgery? | Yes  No |
| 9.If yes, how many day a week ? | Yes  No |
| 10.How many semester with access to robotic surgery did you do ? | (Free answer) |
| 11.Did you performed a complete RATS procedure, as principal operator (considering 75% of completion for a complete procedure) | Yes  No |
| 12.If yes at Q11, which type of procedure ? | Minor surgery (pneumothorax, wedge resection alone)  Mediastinal mass resection  Lobectomy  Segmentectomy  Free comment |
| 13.If yes at Q11, how many? | 1 to 5  6 to 10  11 to 15  16 to 20  > 20 |
| 14. If no at Q11, did you performed a partial RATS procedure (considering when using the master console for any part of the operation). | Yes  No |
| 15. If no at Q14, what was your role in bedside assistant ? | Observation only  Minor assistance (suction, nodes removal…)  Major assistance (vessels or parenchyma stapling…) |
| 16. In your opinion, being fully trained in RATS is | Useless  Usefull  Fundamental |
| 17. Did you performed a wet lab practice on cadaveric or animal model? | Yes  No |
| 18. Are you aware of the existence of a training simulator on the robot console? | Yes  No |
| 19. Did you try the robotic simulator ? | Yes  No |
| 20. If yes at Q19, when | In the OR  In a training facilities  In a congress  In a training sponsored by in industry  Others |
| 21. For how long ? (considering approximatively one hour per session) | < 10h  10 - 20h  20 - 30h  > 30 h |
| 22. Is the access to the robotic simulator sufficient ? | Yes  No |
| 23. What is the appropriate training time needed ?(in hour) | Free answer |
| 24. How would you rate the quality of the robotic training in France? | On a scale from 1 to 5 |
| 25. Do you think you will be autonomous in RATS at the end of you residency ? / As a fellow, do you considered yourself as autonomous in RATS ? | No  Yes  Don’t know |
| 26. Have you ever applied for university degree in robotic surgery? | Yes  No |
| 27. If no, why ? | Not aware of this degree  Too young for that  Not interested  Too expensive  Not enough time  Already trained  Free answer |
| 28. Would you consider experiencing the “RATS Tour”? | Yes / Doing it this year  No  No opinion  Already done / trained |
